# The second European interdisciplinary Ewing sarcoma research summit – A joint effort to deconstructing the multiple layers of a complex disease

**DOI:** 10.18632/oncotarget.6937

**Published:** 2016-01-18

**Authors:** Heinrich Kovar, James Amatruda, Erika Brunet, Stefan Burdach, Florencia Cidre-Aranaz, Enrique de Alava, Uta Dirksen, Wietske van der Ent, Patrick Grohar, Thomas G. P. Grünewald, Lee Helman, Peter Houghton, Kristiina Iljin, Eberhard Korsching, Marc Ladanyi, Elizabeth Lawlor, Stephen Lessnick, Joseph Ludwig, Paul Meltzer, Markus Metzler, Jaume Mora, Richard Moriggl, Takuro Nakamura, Theodore Papamarkou, Branka Radic Sarikas, Francoise Rédini, Guenther H. S. Richter, Claudia Rossig, Keri Schadler, Beat W. Schäfer, Katia Scotlandi, Nathan C. Sheffield, Anang Shelat, Ewa Snaar-Jagalska, Poul Sorensen, Kimberly Stegmaier, Elizabeth Stewart, Alejandro Sweet-Cordero, Karoly Szuhai, Oscar M. Tirado, Franck Tirode, Jeffrey Toretsky, Kalliopi Tsafou, Aykut Üren, Andrei Zinovyev, Olivier Delattre

**Affiliations:** ^1^ Children's Cancer Research Institute, St. Anna Kinderkrebsforschung, Vienna, Austria; ^2^ Department of Pediatrics, Medical University Vienna, Vienna, Austria; ^3^ Departments of Pediatrics, Molecular Biology and Internal Medicine, University of Texas Southwestern Medical Center, Dallas, TX, USA; ^4^ Museum National d'Histoire Naturelle, INSERM U1154, CNRS 7196, Paris, France; ^5^ Children's Cancer Research Center and Department of Pediatrics, Klinikum rechts der Isar, Technical University and Comprehensive Cancer Center Munich (CCCM), Munich, Germany; ^6^ Unidad de Tumores Sólidos Infantiles, Área de Genética Humana, Instituto de Investigación de Enfermedades Raras, Instituto de Salud Carlos III, Madrid, Spain; ^7^ Institute of Biomedicine of Sevilla (IBiS), Virgen del Rocio University Hospital /CSIC/University de Sevilla, Department of Pathology, Seville, Spain; ^8^ University Children's Hospital Muenster, Pediatric Hematology and Oncology, Muenster, Germany; ^9^ INSERM U830, Laboratoire de Génétique et Biologie des Cancers, Institut Curie, Paris, France; ^10^ Institute of Biology, Leiden University, Leiden, The Netherlands; ^11^ Van Andel Institute, Center for Cancer and Cell Biology and Helen DeVos Children's Hospital, Grand Rapids, MI, USA; ^12^ Laboratory for Pediatric Sarcoma Biology, Institute of Pathology of the LMU Munich, Munich, Germany; ^13^ Center for Cancer Rearch, NCI, NIH, Bethesda, MA, USA; ^14^ Greehey Children's Cancer Research Institute, University of Texas Health Science Center, San Antonio, TX, USA; ^15^ VTT Technical Research Centre of Finland Ltd, Espoo, Finland; ^16^ Institute of Bioinformatics, Faculty of Medicine, University of Muenster, Muenster, Germany; ^17^ Department of Pathology and Human Oncology and Pathogenesis Program, Memorial Sloan-Kettering Cancer Center, New York, NY, USA; ^18^ Department of Pediatrics and Department of Pathology, University of Michigan, Ann Arbor, MI, USA; ^19^ Center for Childhood Cancer and Blood Disorders, Nationwide Children's Hospital, and the Division of Pediatric Hematology/Oncology/BMT, The Ohio State University, Columbus, OH, USA; ^20^ Department of Sarcoma Medical Oncology, MD Anderson Cancer Center, Houston, TX, USA; ^21^ Genetics Branch, Center for Cancer Research, National Cancer Institute, Bethesda, MD, USA; ^22^ Pediatric Oncology and Hematology, University Hospital Erlangen, Erlangen, Germany; ^23^ Department of Pediatric Oncology, Sant Joan de Déu Hospital, Barcelona, Spain; ^24^ Ludwig Boltzmann Institute for Cancer Research, Vienna, Austria; ^25^ Institute of Animal Breeding and Genetics, University of Veterinary Medicine and Medical University, Vienna, Austria; ^26^ Division of Carcinogenesis, The Cancer Institute, Japanese Foundation for Cancer Research, Tokyo, Japan; ^27^ University of Glasgow, School of Mathematics and Statistics, Glasgow, UK; ^28^ CeMM Research Center for Molecular Medicine of the Austrian Academy of Sciences, Vienna, Austria; ^29^ INSERM UMR957, Université de Nantes, Nantes, France; ^30^ Department of Pediatrics Research, MD Anderson Cancer Center, Houston, TX, USA; ^31^ Department of Oncology and Children's Research Center, University Children‘s Hospital, Zurich, Switzerland; ^32^ CRS Development of Biomolecular Therapies, Experimental Oncology Lab, Rizzoli Institute, Bologna, Italy; ^33^ Department of Chemical Biology and Therapeutics, St. Jude Children's Research Hospital, Memphis, TN, USA; ^34^ Department of Molecular Oncology, British Columbia Cancer Research Centre, Vancouver, British Columbia, Canada; ^35^ Department of Pediatric Oncology, Dana-Farber Cancer Institute and Boston Children's Hospital, Boston, MA, USA; ^36^ Department of Developmental Neurobiology, St. Jude Children's Research Hospital, Memphis, TN, USA; ^37^ Division of Hematology and Oncology, Department of Pediatrics, Stanford University, Stanford, CA, USA; ^38^ Department of Molecular Cell Biology, Leiden University Medical Center, Leiden, The Netherlands; ^39^ Sarcoma Research Group, Molecular Oncology Laboratory, Bellvitge Biomedical Research Institute (IDIBELL), L'Hospitalet de Llobregat, Barcelona, Spain; ^40^ Department of Oncology, Georgetown University School of Medicine, Washington, DC, USA; ^41^ INSERM, U900, Paris, France; ^42^ Ecole des Mines ParisTech, Fontainbleau, France

**Keywords:** Ewing sarcoma, epigenetics, development, therapy, microenvironment

## Abstract

Despite multimodal treatment, long term outcome for patients with Ewing sarcoma is still poor. The second “European interdisciplinary Ewing sarcoma research summit” assembled a large group of scientific experts in the field to discuss their latest unpublished findings on the way to the identification of novel therapeutic targets and strategies. Ewing sarcoma is characterized by a quiet genome with presence of an *EWSR1*-*ETS* gene rearrangement as the only and defining genetic aberration. RNA-sequencing of recently described Ewing-like sarcomas with variant translocations identified them as biologically distinct diseases. Various presentations adressed mechanisms of EWS-ETS fusion protein activities with a focus on EWS-FLI1. Data were presented shedding light on the molecular underpinnings of genetic permissiveness to this disease uncovering interaction of EWS-FLI1 with recently discovered susceptibility loci. Epigenetic context as a consequence of the interaction between the oncoprotein, cell type, developmental stage, and tissue microenvironment emerged as dominant theme in the discussion of the molecular pathogenesis and inter- and intra-tumor heterogeneity of Ewing sarcoma, and the difficulty to generate animal models faithfully recapitulating the human disease. The problem of preclinical development of biologically targeted therapeutics was discussed and promising perspectives were offered from the study of novel *in vitro* models. Finally, it was concluded that in order to facilitate rapid pre-clinical and clinical development of novel therapies in Ewing sarcoma, the community needs a platform to maintain knowledge of unpublished results, systems and models used in drug testing and to continue the open dialogue initiated at the first two Ewing sarcoma summits.

## INTRODUCTION

**Figure 1:**
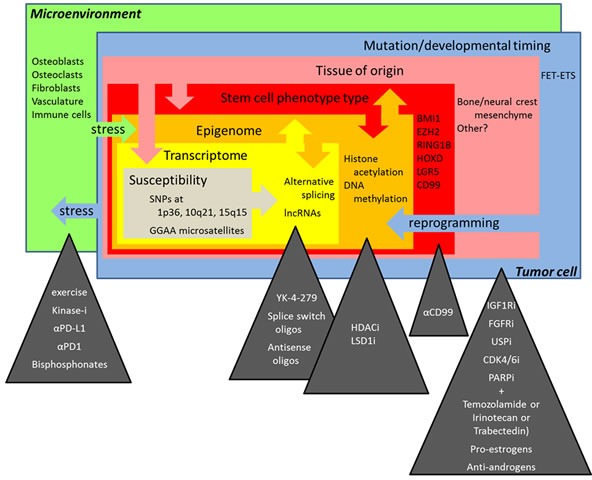
The multiple layers of complexity in Ewing sarcoma biology and novel treatment perspectives discussed at the “Second Interdisciplinary Ewing Sarcoma Research Summit”

**Figure 2:**
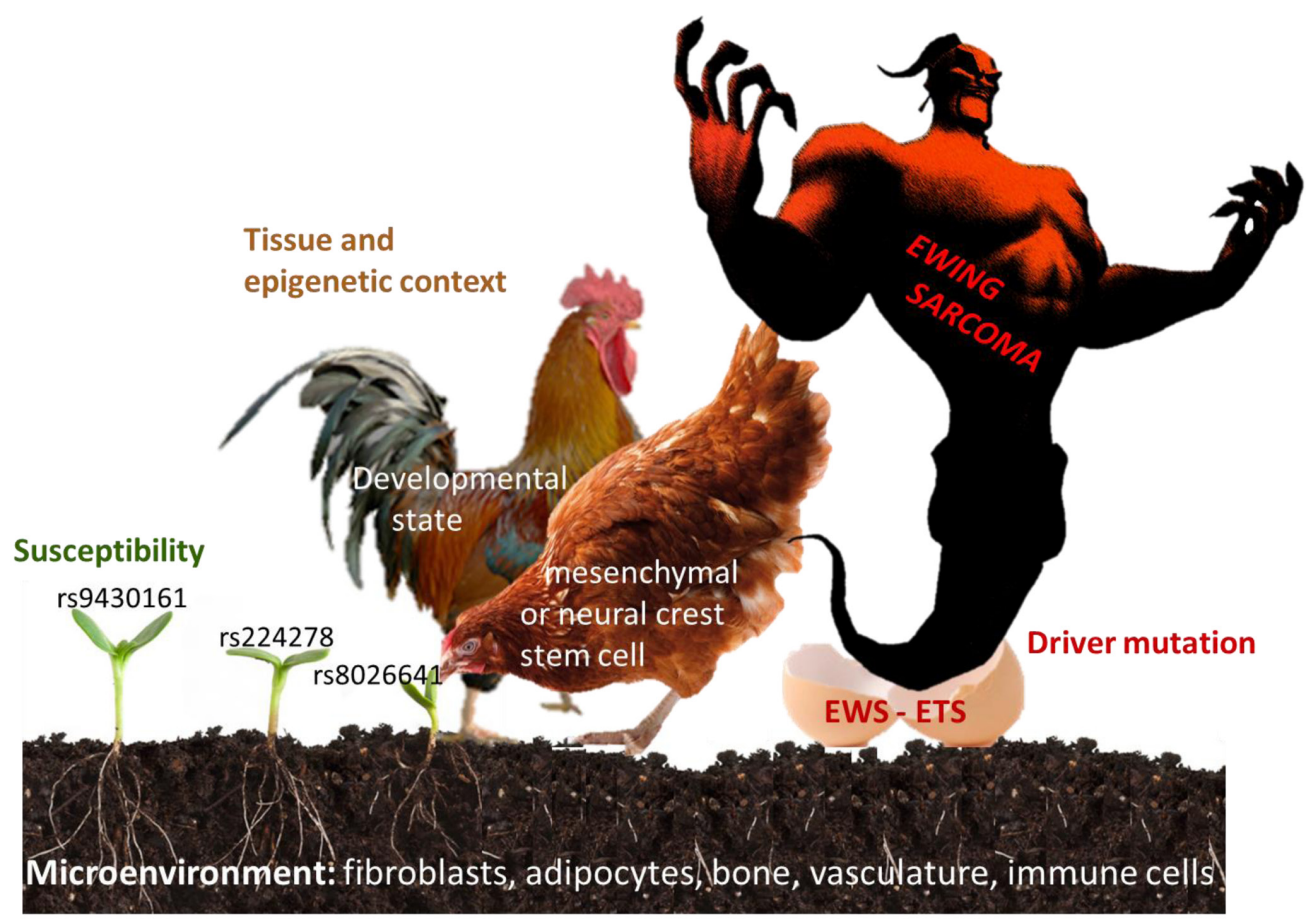
Factors involved in the pathogenesis of Ewing sarcoma Seed (risk alleles at Ewing sarcoma susceptibility loci), soil (tissue microenvironment), hen (tissue of origin), rooster (developmental, epigenetic state), egg (driver mutation), monster (Ewing sarcoma).

Ewing sarcoma is a rare, aggressive cancer of bone and soft tissues that presents most frequently in children and young adults. Progress in Ewing sarcoma therapy has reached a plateau with long-term overall survival rates less than 30% for patients with disseminated disease and 65-75% for patients who present without clinically overt metastases at diagnosis [1]. Using conventional multimodal treatment regimens, only minor improvements in outcome have been achieved during the last 30 years [2]. Therefore, more efficient and specifically targeted approaches are urgently required to combat this deadly disease. Such novel therapeutic strategies are expected to arise from a deeper biological understanding of the pathogenic mechanisms underlying the development, immune escape and metastatic spread of Ewing sarcoma. International Ewing sarcoma research, however, is fragmented and progress is slow due to the rarity of the disease (approximately 3 cases/million/year [3]). Two European framework program 7 (FP7) funded collaborative initiatives therefore put on their agenda activities to overcome this apparent bottleneck, although by different approaches. The research project ASSET (“Assessing and Striking the Sensitivities of Embryonal Tumors”) follows a multi-disciplinary systems biology approach to identify vulnerabilities of the disease. The “European Network for Cancer research in Children and Adolescents” (ENCCA) facilitates and structures networking activities for prioritization of, access to and clinical research on innovative, biologically targeted drugs for the treatment of childhood cancer. However, regular exchange of knowledge and networking is required beyond European borders and beyond the tightly defined agenda of such projects to avoid redundancy and generate synergy in Ewing sarcoma research. In June 2015, four years after the first ENCCA funded Ewing sarcoma meeting [4] and jointly supported by ENCCA and ASSET, the “Second European Interdisciplinary Ewing Sarcoma Research Summit” assembled 77 researchers from Europe, Japan, the US and Canada to share exclusively unpublished results and to discuss future research directions and opportunities for clinical translation (Suppl. Table 1 for list of participants). It was the largest purely scientific Ewing sarcoma convention so far. Maybe it was the spirit of the venue at Institute Curie in Paris, where the *EWSR1-FLI1* fusion gene was discovered as the defining marker and driver of the disease almost 25 years ago, combined with the unique meeting format, that made it highly successful in bringing together colleagues and competitors in the field, fostering trustful exchange, open discussion, and the initiation of new promising collaborations. This review summarizes some exciting new insights into Ewing sarcoma biology presented at this meeting (Figure 1). Since speakers frequently used metaphors in their presentations, the chapters of this review are named accordingly (Figure 2).

## THE MONSTER

In our nightmares and in horror movies, it is the unknown, the mysterious that threatens us in the shape of a dangerous monster. Ewing sarcoma remains such a monster, a “genetically engineered monster” with the *EWSR1-ETS* gene fusion as the major driver of its nefarious activities, as Paul Meltzer pointed out while introducing the topic of “Spatial and temporal genetic and non-genetic diversity of Ewing sarcoma” (Table 1). From recent sequencing studies it has become clear that with the exception of the well-known *EWSR1-ETS* gene fusions, which drive a complex tumor specific transcriptional program, the Ewing sarcoma genome is relatively quiet [5-9]. Franck Tirode provided an overview on the molecular heterogeneity of Ewing and Ewing-like tumors in a cohort of 130 sarcomas. By RNA sequencing he demonstrated that tumors with *FET-ETS* gene fusions involving *EWSR1* or *FUS* with members of the *ERG* (*FLI1, ERG*) or *PEA3* (*ETV1,* ETV4) subfamily of *ETS* transcription factor genes cluster tightly together in a homogenous group, separate from Ewing-like sarcomas with *EWSR1* fusions to non-*ETS* genes (i.e. *NFATC, POU5F1, SMARCA5*) and from those harboring the recently described *BCOR-CCNB3* or *CIC-DUX4* gene fusions.

**Table 1:** Genetic and non-genetic sources of inter- and intra-tumor heterogeneity in Ewing sarcoma

Genetic	Germline genetic risk (susceptibility loci on chromosomes 1, 10, 15; metastasis loci?)
	Different EWSR1-transcription factor fusions
	Copy number variations (i.e. gains on chromosomes 8, 12, 1q; loss at 16p)
	Clonal complexity of tumor
	Therapy driven mutation/selection
Non-genetic	Plasticity of tumor/tumor stem cells
	Heterogenous epigenetic states (chromatin factors and DNA methylation)
	RNA metabolism (splicing, editing, degradation)
	Activity of non-coding RNAs (microRNAs, long noncoding RNAs, others)
	Metabolism (tissue site, microenvironment, stress and therapy driven)
	Proliferative states (dormancy)
	Micro-environment modification of tumor cells (i.e. immune system)
	Age and gender

Some of the mystery behind Ewing sarcoma pathogenesis and the inter-patient heterogeneity in its response to treatment may arise from non-genetic sources, such as the epigenome. Paul Meltzer stressed that the epigenetic states of cancer are generally abnormal, not fitting any healthy tissue, and thus, cancers deviate from physiologic epigenetic programs. In Ewing sarcoma, widespread epigenetic rewiring of gene regulatory regions was recently demonstrated to be induced by EWS-FLI11 [10, 11]. Knowledge about the exact mechanisms of epigenetic dysregulation may provide novel therapeutic opportunities. Stephen Lessnick reported on the preclinical testing of HCI2577, a second generation lysyl specific demethylase (LSD1) inhibitor, which they found to reverse the EWS-FLI1 transcriptional signature to a large extent and to cause apoptosis. However, based on previous results of the group on the anti-tumor effects of the first generation inhibitor HCI2509 [12], LSD1 inhibition affected both EWS-FLI1 activated and repressed genes that are bound by the fusion oncogene, the exact mechanism of functional interaction between EWS-FLI1 and LSD1 remains obscure.

Non-genetic variability may also be the basis for distinct treatment sensitivity. Paul Meltzer reported on differences in tumor transcriptomes of patients transiently responding or not to the R1507 IGF1R antibody in the SARC-011 trial. The molecular basis for resistance development may lie in genetic or non-genetic intra-tumor heterogeneity. Olivier Delattre introduced single cell transcriptome analysis to study variation and transitions in transcriptional programs in a population of Ewing sarcoma cells with fluctuations in EWS-FLI1 expression. Nathan Sheffield demonstrated the power of reduced representation bisulfite sequencing (RRBS) to study cell-to-cell heterogeneity in DNA methylation of a tumor. Neighboring CpG sites in a given genomic region often show concordant methylation status, but the methylation of neighboring CpGs is occasionally not matching, leading to potentially disordered methylation patterns. Because each read may span several methylation sites and is derived from the sequencing of one DNA molecule, the comparison between methylation status at neighboring CpGs within a read can be used to assess intra-tumor heterogeneity. Nathan Sheffield demonstrated this phenomenon in a cohort of 140 Ewing sarcomas and normal mesenchymal stem cell samples, which are currently being explored for prognostic methylation patterns in a collaborative project between Austria, France and Germany.

Comparative methylation profiling between Ewing sarcoma and normal tissues also has the potential to uncover unknown tumor specific molecular traits. Using the Infinium 450K methylation array to study promoter methylation, Oscar Tirado's group identified Ewing sarcoma specific inactivation of the *PTRF*/*Cavin-1* gene, which, when co-expressed with the EWS-FLI1 activated target gene Caveolin-1 (*CAV1*), induced TP53 dependent cell death. These results support the use of demethylating drugs for the treatment of Ewing sarcoma.

High-throughput sequencing technologies enable characterization of the genetic and epigenetic make-up and the transcriptional signature of tumors, and, by correlation analysis, how the one affects the other. Andrei Zinovyev described a computational method based on a Boolean mathematical model to predict genetic interactions and thus explain deviations of the phenotypic quantitative effect of multiple gene mutations from their simple additive effect as applied to Ewing sarcoma [13]. Theo Papamarkou discussed a novel mathematical method to also integrate so far poorly investigated RNA editing effects in gene regulatory networks based on RNA-seq data.

Several presentations addressed approaches to follow the tracks of the monster over time. Markus Metzler and Brian Crompton described methods for non-invasive detection of impending relapse based on the high-sensitivity detection of circulating tumor DNA in the plasma of Ewing sarcoma patients. Long-range PCR and capture sequencing methods are being applied to determine patient-specific genomic *EWSR1-FLI1* breakpoints which are subsequently used to detect tumor DNA shed in the circulation by digital PCR. Thus, Uta Dirksen presented first results from the “EFACT” (*EWSR1-FLI1* sequence analysis from ctDNA) study ancillary to the European clinical Ewing sarcoma trials Ewing2008 and Ewing 2012, while Marc Ladanyi presented data based on the MSK-IMPACT program at Memorial Sloan Kettering Cancer Center, which provided proof of principle for detection of circulating tumor (ct) DNA in the plasma preceding clinically overt relapse of Ewing sarcoma.

## HEN AND EGG

While *Omics* technologies take a scan of Ewing sarcoma, the fully matured monster, and allow us to monitor its variability and plasticity, the egg from which it hatched remains unknown. Much of the difficulty in identifying the tissue of origin for Ewing sarcoma arises from the toxicity of *EWSR1-ETS* fusion genes to most cell types. This is probably the reason why most attempts to generate animal models of EWS-ETS driven oncogenesis have so far failed. The current view is that the disease arises from some mesenchymal or neural crest derived stem or progenitor cell. Erika Brunet reported that though it was possible to induce the *EWSR1-FLI1* gene rearrangement in adult mesenchymal stem cells by zinc finger nucleases or CRISPR/Cas9 mediated gene editing, the gene fusion was unstable and gradually counter-selected in this cell type. Using the latter genome editing approach, Marc Ladanyi also showed efficient induction of the *EWSR1-FLI1* gene rearrangement in HEK293 cells. Aykut Üren summarized a plethora of mostly unpublished (because unsuccessful) attempts from various labs to generate *EWS-ETS* transgenic tumor models in rodents and fish by either conditional tissue specific activation or topical administration of the fusion gene. Most of them led to no phenotype or embryonic lethality, tissue damage (i.e cardiomyopathy [14]) and/or developmental defects, but not tumorigenesis. These studies included expression of the gene fusion in osteoblast precursors, limb bud mesenchyme, neuronal tissue, or muscle, using a variety of promoters to drive the gene fusion (CMV, LTR, EWSR1, Rosa26, Pgk, Nse, dNEFL, TRE) and Cre lines to activate the transgene (Runx2Cre, OsxCre, Prx1Cre, Dermo1Cre, P0Cre, Col1a2Cre, Sox9Cre). Given that even in humans Ewing sarcoma incidence varies with ethnicity (the disease is particularly rare in Africans), it is possible that model organisms are not susceptible to this disease. Aykut Üren discussed several potential reasons for this assumption, including different gene splicing patterns and variations in the lengths of GGAA micro satellites, two features that may differentially affect EWS-FLI1 function and target gene expression in mice and men.

However, Richard Moriggl presented data from a Prx1Cre driven EWS-FLI1 mouse model established in his group, which implicates developmental timing of the gene fusion as an important factor in tumorigenesis. When activated (using a tamoxifen-inducible Cre recombinase) in a narrow time window around birth, EWS-FLI1 expression in the bone mesenchyme resulted in sarcomas that recapitulate the human Ewing sarcoma EWS-FLI1 transcriptional signature. Consistent with this finding, Takuro Nakamura described their recently published transplantation model of tumors arising from EWS-FLI1 transgenic embryonic superficial zone cells from the articular cartilage at an ERG and PTHLH expressing developmental stage [15], and compared it to an unpublished similarly constructed model of CIC-DUX4 induced tumorigenesis from the same cell type. He demonstrated that the *CIC-DUX4* gene fusion activates PEA3 and ERG family ETS transcription factors and, similar to EWS-FLI1, down-regulates apoptosis genes.

In zebrafish, James Amatruda reported on the use of transgenic EWS-FLI1 zebrafish for small-molecule and genetic screens, and a new model of CIC-DUX4-Ewing-like sarcomas. Wietske van der Ent discussed their attempts to generate a flexible transgenic zebrafish model of EWS-ERG induced tumorigenesis using the binary UAS/GAL4 system. Similar to mice, most tested fish tissues did not tolerate the fusion gene with one exception: Expression of GFP-tagged EWS-ERG in neuronal tissues led to developmentally impaired embryos with large amounts of GFP-positive transformed cells, which showed histological features similar to Ewing sarcoma. Transcriptomic and proteomic analysis of regulated gene expression in these embryos showed that there was an overlap with human and murine expression profiles linked to Ewing sarcoma development.

The notion that certain embryonal mesenchymal and neuronal tissues tolerate EWS-ETS gene expression in animal models, at least at defined developmental stages, is consistent with the current view that Ewing sarcoma arises from some potentially neural crest-derived mesenchymal or neuronal progenitor cells. Elizabeth Lawlor reviewed the published evidence for this hypothesis and put it into context with available knowledge on pluripotent adult stem cells. These cells express high levels of polycomb proteins to suppress a large number of differentiation genes. Among them are *EZH2* and *BMI-1* and, as Inmaculada Hernandez and Jaume Mora reported, *RING1B* (*RNF2*), which affects genes of heme biosynthesis, endothelial and neural development, and which they found to protect Ewing sarcoma cells from NFκB induced apoptosis through regulation of the sodium channel NaV1.6. Summarizing available evidence, Elizabeth Lawlor speculated that BMI-1 positive cells may provide the permissive environment for fusion gene activity, as has previously been reported for E2A-PBX1 in hematopoietic stem cells [16], and more recently demonstrated by her own group for EWS-FLI1 in neural crest derived stem cells (NCSC) [17]. The chimeric oncogene would then perpetuate the progenitor-like state by hijacking the developmental transcription program.

On the other hand, there are developmental genes which escape suppression by overexpressed polycomb proteins in Ewing sarcoma. It has previously been demonstrated that posterior homeobox (HOX) cluster D genes are paradoxically highly expressed in this disease [18]. Günther Richter and Stefan Burdach studied the mechanism and consequences of this up-regulation. They presented preliminary data indicating that *HOXD10,11* and *13* genes are regulated via the EWS-FLI1 target DKK2, presumably involving the canonical WNT/β catenin pathway, to drive chondrogenic but not osteogenic differentiation. Knockdown of *HOXD* genes resulted in down-regulation of *RUNX2*, *PTHLH*, *BGLAP*, *PDGF*-*BB* and *MMP1* reducing contact independent growth and metastatic potential of Ewing sarcoma cells. Elizabeth Lawlor concluded that, when it comes to the hen and egg question of which is the limiting factor in Ewing sarcomagenesis, cell type/stage of origin or EWS-ETS activity, the two features are tightly intertwined and cannot be separated from each other.

## SEED AND SOIL

In contrast to infectious diseases, where the affected patient's tissues serve as host for the invading pathogens, cancer constitutes a host tissue in itself. It is, however, comprised of multiple cell types that influence each other, as Lee Helman pointed out in his introduction to the topic “Targeting the Ewing sarcoma ecosystem”. Tumor-microenvironment interactions occur between multiple host cell types. Tumor infiltrating fibroblasts, adipocytes and immune cells alter their metabolism to adapt to the tumor environment where they create an immunosuppressive milieu. Lee Helman referred to a study that demonstrated the importance of the mitochondrial Krebs cycle in shaping the nuclear methylome of several cancers [19] and speculated that metabolic adaptations in tumor-microenvironment interactions may affect the epigenome of both tumor and infiltrating host cells. In addition, he pointed out that genotoxic treatments elicit DNA damage responses in healthy tissues leading to inflammatory responses that affect tumor cells and metastases. Steve Lessnick discussed the contrasting effects of normoxia and hypoxia on proteolytic cleavage and isoform expression of neuropeptide Y (NPY) with opposite consequences for cell proliferation and apoptosis (Tilan et al. 2013). Consequently, several talks addressed the interaction between the seed - the molecular underpinnings of Ewing sarcoma, and the soil - the tumor microenvironment.

Previously, the group of Franck Tirode demonstrated that sustained knockdown of EWS-FLI1 restores multipotency to Ewing sarcoma cell lines *in vitro* [20]. Expanding on this observation, Olivier Delattre reported that EWS-FLI1 silencing in the *in vivo* context of a mouse xenograft model resulted specifically in adipogenic differentiation of tumor cells. Similarly, Patrick Grohar observed replacement of the tumor tissue in Ewing sarcoma bearing mice by fat tissue of human origin upon treatment with PM01183, a second generation Trabectedin analog. This drug, which similar to UV distorts and breaks DNA strands, seems to inactivate EWS-FLI1 by driving it into the nucleolus. Thus, it appears that the tumor microenvironment has a profound effect on the differentiation route of Ewing sarcoma cells upon inactivation of the fusion gene *in vivo*. However, it is not clear if this effect resulted from the direct differentiation of Ewing sarcoma cells or due to the creation of a pro-adipogenic microenvironment.

Wnt/β-catenin signaling is one of the developmental pathways deregulated in Ewing sarcoma. In mesenchymal tumors it acts as a morphogen rather than driving proliferation. It has been demonstrated previously that activation of the Wnt pathway in Ewing sarcoma is potentiated by R-spondins, the ligands of the somatic stem cell surface receptor LGR5, which stabilizes β-catenin and functions as an oncogene in several human cancers [21]. Elizabeth Lawlor reported that while under standard *in vitro* growth conditions Wnt signaling is off and can only be activated upon EWS-FLI1 silencing and ligand addition, focal β-catenin staining is observed in primary tumors. Because LEF1/TCF is a transcriptional downstream effector of Wnt/ β-catenin signaling and its expression is associated with poor prognosis, Elizabeth Lawlor speculated about intra-tumor heterogeneity of EWS-FLI1 expression and the role of the tumor micro-environment in activating Wnt/ β-catenin and its role in promoting tumor cell motility and metastasis. In this context, the applicability of the tumor stem cell model to Ewing sarcoma was discussed by Eberhard Korsching.

In light of recent evidence that suggests the EWS-FLI fusion protein may act as a pioneer factor capable of eliciting broad sweeping epigenetic effects, novel *ex vivo* models may provide an innovative platform to determine how microenvironmental cues—and their downstream signaling cascades—contribute to and/or reinforce epigenetic changes linked to the aberrant EWS-FLI fusion. One way to study bidirectional feedback mechanisms that exist between Ewing sarcoma and its adjacent microenvironment is with *ex vivo* tissue engineered three-dimensional culture models. Such a model was pioneered by Joseph Ludwig's laboratory. As recently published, in sharp contrast to Ewing sarcoma cells cultured upon flat tissue culture plastic ware, cells cultured on biologically inert poly(ε-caprolactone) (PCL) microfiber scaffolds placed within a flow perfusion bioreactor were hypersensitive to IGF-1R targeted monoclonal antibodies, which represent a promising class of precision-guided experimental drugs currently in evaluation in early phase clinical trials. Intriguingly, though improved nutrient delivery throughout the porous scaffolds contributed to cell survival within this preclinical model, heightened sensitivity to IGF-1R antibodies seemed to be mediated by physiological levels of shear stress that can be precisely regulated experimentally [22]. Similarly, Francoise Rédini used novel mineralized scaffolds to investigate the vicious cycle between osteoclasts, bone stromal cells/osteoblasts and tumor cells in Ewing sarcoma progression by transcriptomic analysis. Results are integrated with differential gene expression patterns and therapy responses observed in patients and pre-clinical models of bone versus soft tissue Ewing sarcoma. Such an approach may also be useful to validate the potential influence of the stromal component on the prognostic transcriptional signature of Ewing sarcoma with respect to chemokine receptor expression CXCR7 and CXCR4 isoforms that differ in their affinity to antagonistic ligands CXCL12 and CXCL14, discussed by Karoly Szuhai.

Tumor growth beyond the reach of existing vasculature triggers cellular adaptations to overcome limiting nutrient and oxygen delivery. In addition, oncogenic activation and metabolic re-programming elicit cell intrinsic stresses. Under these conditions, metabolism is re-wired to support cellular energy homeostasis and to supply the building blocks for biomass [23]. One mechanism of energy preservation is selective withdrawal of mRNAs from translation by storage in ribonucleoprotein complexes, called stress granules. Poul Sorensen described that under oxidative stress up to 60% of mRNA gets entrapped in stress granules. He recently reported that the RNA binding protein YB-1 translationally activates expression of a number of stress-responsive proteins including HIF1α [24], and activates translation of the stress granule nucleator, G3BP1 [25]. He reported on proteomic approaches to define the composition of YB-1 containing stress granules, and the promising activity of several histone deacetylase inhibitors in preventing stress granule formation.

A so far unrecognized hint to a potential role of stress granules in the pathogenesis of Ewing sarcoma may arise from the functional analysis of genes in the vicinity of Ewing sarcoma susceptibility loci. A previously published genome-wide association study from the Delattre lab identified candidate risk loci on chromosomes 1, 10, and 15 [26]. The chromosome 1 susceptibility SNP rs9430161 is located in the vicinity of *TARDBP*, encoding an RNA binding protein that is structurally similar to and co-localizes with FUS and EWS to stress granules. Heinrich Kovar now reported on preliminary results generated by Dave Aryee suggesting that the chromosome 15 associated risk locus rs4924410 may affect the activity of a further stress granule associated protein, SRP14, via EWS-FLI1 dependent regulation of a novel long non-coding (lnc)RNA.

While these studies are still in their infancy, Thomas Grünewald summarized his recently completed study on the functional analysis of the chromosome 10 encoded Ewing sarcoma susceptibility locus, which he demonstrated to affect expression of the Ewing sarcoma growth regulatory *EGR2* gene through extension of an EWS-FLI1 bound enhancer-like GGAA microsatellite [27].

All together, these findings underline the context-specific role of EWS-ETS proteins in giving birth to the monster. It is clear that dysregulation of transcriptional programs by EWS-ETS driven wide-spread enhancer reprograming and promoter deregulation is a major factor shaping the biological and clinical characteristics of this monstrous disease. At the meeting, however, some less well characterized EWS-ETS activities and their potential roles in sarcomagenesis and progression were discussed.

Alejandro Sweet-Cordero reported that EWS-FLI1 deregulates the expression of >300 lncRNAs, some directly some indirectly. He presented data on two highly expressed Ewing sarcoma specific, EWS-FLI1 regulated lncRNAs, EWSAT1 [28] and EWSAT2 (lnc659). He demonstrated that lncRNA EWSAT1 is involved in EWS-FLI1 mediated gene repression whereas ongoing studies are directed at identifying the mechanism of EWSAT2. Importantly, knock-down of both lncRNAs interferes with tumor cell growth *in vitro* and *in vivo*.

## TAMING THE MONSTER

To identify vulnerabilities of Ewing sarcoma, Kimberly Stegmaier presented the “Pediatric cancer dependencies project”, which combines high-throughput shRNA and drug screens, super-enhancer profiling, and CRISPR/Cas9 mediated knockout on a multitude of cell lines *in vitro*. Using this approach, they identified the regulatory subunit of the protein phosphatase PP2A complex, STRN4, and the cyclin dependent kinase CDK4 as essential for Ewing sarcoma cell growth/survival. In fact, they found that the CDK4/6 inhibitor LEE011 (Novartis) has promising *in vitro* and *in vivo* cytostatic and cytotoxic activity on Ewing sarcoma cells [29].

Branka Radic Sarikas performed a synergy screen on selected FDA approved drugs and identified synergistic cytotoxic effects of IGF1R and protein kinase C inhibition in the presence of EWS-FLI1. Kristiina Iljin reported on the results of a drugable siRNA cell viability screen in an inducible EWS-FLI1 shRNA Ewing sarcoma cell line interrogating nearly 7000 genes with 4 siRNAs per gene for EWS-FLI1 dependencies, which was performed as part of the “ASSET” project. In addition, they also performed a small compound screen of >3000 agents comparing EWS-FLI1 on/off states in the same model. Integrating a variety of genomic data sets and mining the literature Kalliopi Tsafou developed an algorithm to link drug effects to genes. By this approach, she identified nodes for which several drugs scored high in the synthetic lethality screen with EWS-FLI1 expression in the Ewing sarcoma cell line A673. Among top hits were histone deacetylases. Consistent with this finding, Anang Shelat and Elizabeth Stewart reported exquisite sensitivity of Ewing sarcoma cells to the class I selective HDAC inhibitor OKI-5.

They also reported that adding a poly(ADP-ribose)polymerase inhibitor such as Talazoparib or Olaparib to Irinotecan and dose-escalating Temozolomide yielded approximately 90% survival in Ewing sarcoma xenografted mice compared to 100% mortality in mice receiving Irinotecan and full-dose Temozolomide. Refering to a recently published study, they discussed the expression effect of the EWS-FLI1 target *SLFN11* on sensitivity to this drug combination [30]. Synergistic activity for combination treatment of patient derived xenografts with Olaparib and Trabectedin was reported by Enrique de Alava [31]. While PARP1 was previously demonstrated to regulate EWS-FLI1 expression and transcriptional activity [32], and Trabectidin reported to revert the EWS-FLI1 transcriptional signature [33], the de Alava study did not detect any effect of the Olaparib plus Trabectedin combination on EWS-FLI1 target gene expression at Trabectedin concentrations 5-10x lower than previously reported to suppress EWS-FLI1.

In addition to targeting hubs in the EWS-ETS downstream gene regulatory network, perturbation of the expression or functional activity of the gene fusion product itself is considered the holy grail from which innovative Ewing sarcoma specific therapies may arise. A siRNA screen performed in Lee Helman's lab to identify genes whose depletion recapitulates the transcriptional effects of EWS-FLI1 knockdown, identified several components of the splicing machinery. In fact, knockdown of one of them, HNRNPH1, perturbed the correct splicing of primary EWS-FLI1 transcripts in cells with breakpoints in *EWSR1* intron 8 leading to an out-of-frame fusion product. An alternative approach to disrupt correct EWS-FLI1 RNA processing was presented by Marc Ladanyi, who showed *in vitro* data on treatment with splice-switching oligonucleotides to introduce premature polyadenylation from internal polyA sites of the fusion RNA. In addition, Jeff Toretsky's group recently demonstrated that, in turn, altered RNA splicing is one of the EWS-ETS fusion protein's oncogenic functions, which can be inhibited by the small molecule YK-4-279 [34]. Jeff Toretsky discussed the difficulty of pharmacologically targeting the EWS-ETS fusion protein introducing the concept of protein concentration dependent physical phase separation, potentially nucleated by local enrichment at GGAA microsatellites [35]. Although so far all attempts to map the exact binding site of the YK-4-279 compound along the fusion protein failed, and no influence on the EWS-FLI1 transcriptional signature was observed, results presented by Lee Helman and Jeff Toretsky encourage clinical evaluation of splicing inhibitors in Ewing sarcoma patients.

Alternatively, targeting EWS-ETS protein stability may provide a so far unexplored therapeutic option. EWS-FLI1 stability is regulated by K48 polyubiquitinylation and proteasomal degradation. Using a targeted shRNA screen interrogating 21 Ewing sarcoma expressed deubiquitinating enzymes, Beat Schäfer's group identified ubiquitin-specific protease USP19 as an EWS-FLI1 regulatory enzyme, whose knockdown destabilizes EWS-FLI1 protein and may therefore serve as an attractive therapeutic target.

Pre-clinical drug validation requires studies in model organisms. In the absence of validated rodent models for most pediatric cancers, the “Pediatric Preclinical Testing Program” (PPTP) studied 67 drugs on 83 different xenograft mouse models, in all together 2134 drug/model comparisons. Peter Houghton reported that retrospective analysis of the results for any of 1000 randomly selected mice accurately predicted the response of the whole group in each comparison in 75% of cases. The predictive power of the response of a single mouse increased to 95%, if one deviation per group was allowed. Based on these results, he provocatively suggested to use a single mouse xenograft instead of 10 mice per patient sample or cell line in future pre-clinical drug efficacy tests. This strategy would lower costs and increase throughput, two key factors in *in vivo* drug screens. An attractive alternative to mice in this respect are zebrafish. James Amatruda presented a chemical suppressor screen in a *mitfa:EWS-FLI1* transgenic zebrafish model. EWS-FLI1 is well tolerated by melanocytes which increase in number due to oncogene expression. Drug-induced reduction in melanocytes can therefore be used as a surrogate readout for activity against EWS-FLI1 driven cell proliferation. Testing 1200 compounds, they identified activity for several kinase inhibitors, bisphosphonates and, interestingly, pro-estrogens and anti-androgens.

For an anti-cancer drug to be effective, it needs to reach its target via the blood stream. However, about 50% of tumor vessels are non-functional. Therefore normalization of the tumor vasculature should improve drug delivery. Keri Schadler reported that vascular shear stress, which is induced in endothelial cells by the blood flow in response to aerobic exercise, induces functional tumor vasculature and increases chemotherapeutic efficacy, as exemplified for doxorubicin. She presented data identifying a role for nuclear factor of activated T cells (NFAT) c1 and thrombospondin TSP1 in the normalization of the tumor vasculature.

Whole genome sequencing technologies have provided ultimate proof that cancers are vastly different from normal tissues and that some of these differences will be recognized by the immune system if immune checkpoints can be overcome. Identification of mechanisms by which tumor cells manipulate the immune system is of critical importance for developing strategies that reverse tumor-induced immunosuppression and sensitize tumor cells to lysis by preexisting or therapeutic effector cells. Cellular imunotherapies for Ewing sarcoma are under development but have not yet been effective. In many cancers, the number of mutations predicts response to checkpoint targeting drugs (i.e. anti-PDL1 and -PD1 antibodies). Since the mutational landscape of Ewing sarcoma is relatively quiet, the question arises if this type of cancer is sensitive to immunotherapy. As a first step to address this problem, Claudia Rössig reported preliminary results on local expression of the immune-inhibitory ligand PD-L1 and the non-classical HLA molecule HLA-G in the Ewing sarcoma microenvironment, as determined by immunohistochemistry in pre-therapy tumor biopsies.

In addition to the *EWSR1-ETS* gene fusion, Ewing sarcoma is characterized by high CD99 expression. Katia Scotlandi explored the therapeutic potential of targeting this enigmatic surface glycoprotein. She presented new results on non-apoptotic tumor cell killing by the murine monoclonal antibody O662 and a human, CD99-directed single chain antibody. This type of cell death is initiated by HRAS and RAC-1 activation and dysregulation of micropinocytosis, and is insensitive to overexpression of anti-apoptotic Bcl2 family members and ERK activation. Dysregulation of RAS signaling in Ewing sarcoma may also be deduced from work presented by Florencia Cidre-Aranaz. She observed EWS-FLI1 mediated suppression of the fibroblast growth factor receptor (FGFR) antagonist sprouty 1 (SPRY1). FGFR1 has been recently demonstrated to be active in Ewing sarcoma [9]. SPRY1 antagonizes ERK activation of RAS and acts as a tumor suppressor in Ewing sarcoma cells reducing proliferation and migration when ectopically overexpressed. Florencia Cidre-Aranaz reported that increased SPRY1 expression was associated with a better relapse-free and overall survival, while low SPRY1 levels associated with increased metastasis in patients. These data may provide a rationale to consider therapeutic use of FGFR1 and RAS inhibitors in the treatment of Ewing sarcoma.

## PROGRESS SINCE THE FIRST INTERDISCIPLINARY EWING SARCOMA RESEARCH SUMMIT

In-depth genome and transcriptome sequencing studies identified widespread dynamic inter- and intra- tumor heterogeneity of Ewing sarcoma down to the single cell level. The rapid expansion and spread of sophisticated novel next generation sequencing applications beyond RNA and genome analysis have provided unprecedented insights into chromatin dynamics. Most importantly, it has become clear that reprogramming of the epigenome and alternative RNA splicing downstream of EWS-FLI1 play central roles in Ewing sarcoma pathogenesis and may therefore provide novel therapeutic targets. As the epigenome serves the ultimate “receptor” for developmental and microenvironmental signaling cues, we have started to understand how tissue context, architecture, and metabolic state may influence tumor growth with implications for therapy response. For the first time, a mouse model of Ewing sarcoma is on the horizon based on developmentally tightly timed EWS-FLI1 expression in the bone mesenchyme, which has the potential of speeding-up preclinical drug development in the near future.

## CONCLUSIONS

Ewing sarcoma remains a monstrous disease to patients, families, doctors and scientists. It hatches from the malicious activities of EWS-ETS fusion proteins as the egg, bred by some neural crest or mesenchyme derived stem cell at a defined developmental stage, as parental hen and rooster. Researchers at the ASSET/ENCCA meeting discussed the role of the soil - the microenvironment, and the seed - a susceptible genetic background, which are required to feed the chick to become the monster that is so difficult to tame. Laboratory data, both mature and preliminary, were presented in support of new treatment concepts in the war against Ewing sarcoma, including the use of epigenetic and specific pathway-directed drugs targeting the tumor and its microenvironment (Figure 1). To enable the next step along the path to clinical translation of these promising insights, pre-clinical compound testing in animal and/or 3D culture systems, it was recognized that the field would greatly benefit from an exchange platform to allow for sharing of cell lines and models, omic and linked clinical data, standard operating procedures and harmonization of protocols. The group agreed that the atmosphere of trust, openness and cooperativeness demonstrated at this meeting should facilitate the establishment of an international working group to put in place a common database that keeps memory of tested compounds and systems. This would be required for efficient prioritization of novel drugs for further pre-clinical and clinical development, which may hopefully lead to a major transition in the way patients with Ewing sarcoma are treated. Such a working group should also involve patient advocacy groups in the hope that they may help obtain sustained funding sources for this endeavor. With their support, ASSET and ENCCA tried to pave the way, but with termination of these projects in 2016, novel funding strategies are needed to keep up the fruitful momentum of the “Second European Interdisciplinary Ewing Sarcoma Research Summit”.

## SUPPLEMENTARY MATERIAL TABLE


