# Supplementary material for: The second European interdisciplinary Ewing sarcoma research summit – A joint effort to deconstructing the multiple layers of a complex disease
Source: Oncotarget. 2016 Jan 18;7(8):8613–24. doi: 10.18632/oncotarget.6937 (PMC4890991; doi:10.18632/oncotarget.6937)
Supplement: Supplementary file 1 [file oncotarget-07-8613-s001.pdf]

The second European interdisciplinary Ewing sarcoma research summit – A joint effort to deconstructing the multiple layers of a complex disease

Supplementary Material

Supplementary Table

2nd European Ewing sarcoma interdisciplinary research summit participants (in alphabetical order):

Javier Alonso
James Amatruda
Dave Aryee
Erika Brunet
Stefan Burdach
Florencia Cidre
Brian Crompton
Enrique de Alava
Olivier Delattre
Uta Dirksen
Nathalie Gaspar
Chiara Giorgi
Patrick Grohar
Thomas Grünewald
Wolfgang Hartmann
Lee Helman
Inmaculada Hernandez
David Herrero-Martin
Peter Houghton
Kristiina Iljin
Camille Jacques
Javaheri Tahereh
Eberhard Korsching
Heinrich Kovar
Raushan Kurmasheva
Marc Ladanyi
Eve Lepouble
Elizabeth Lawlor
Stephen Lessnick
Ivo Leuschner
Laura Lopez Garcia
Joseph Ludwig
Perrine Marec-Berard
Oscar Martinez-Tirado
Paul Meltzer
Ariadna Mendoza
Markus Metzler
Jean Michon
Jaume Mora
Richard Moriggl
Takuro Nakamura
José Luis Ordónez
Benjamin Ory
Theodore Papmarkou
Pierro Picci
Gaelle Pierron
Jenny Potratz
Branka Radic Sarikas
Francoise Redini
Günther Richter
Claudia Rössig
Keri Schadler
Beat Schäfer
Katia Scotlandi
Nathan Sheffield
Anang Shelat
Neal Shukla
Ewa Snaar-Jagalska
Poul Sorensen
Kimberly Stegmaier
Elizabeth Steward
Sandra Strauss
Didier Surdez
Alejandro Sweet-Cordero
Karoly Szuhai
Roberto Tirabosco
Franck Tirode
Eleni Tomazou
Jeffrey Toretsky
Kalliopi Tsafou
Rebekka Unland
Aykut Üren
Wietske van der Ent
Joshua Waterfall
Sarah Watson
Andrei Zinovyev
Stefan Zöllner
